# Effects of Hand and Hemispace on Multisensory Integration of Hand Position and Visual Feedback

**DOI:** 10.3389/fpsyg.2019.00237

**Published:** 2019-02-12

**Authors:** Miya K. Rand, Herbert Heuer

**Affiliations:** Leibniz Research Centre for Working Environment and Human Factors, TU Dortmund (IfADo), Dortmund, Germany

**Keywords:** reaching, tool use, sensory integration, implicit measure, explicit measure, dominant hand, nondominant hand, hemispace

## Abstract

The brain generally integrates a multitude of sensory signals to form a unified percept. Even in cursor control tasks, such as reaching while looking at rotated visual feedback on a monitor, visual information on cursor position and proprioceptive information on hand position are partially integrated (sensory coupling), resulting in mutual biases of the perceived positions of cursor and hand. Previous studies showed that the strength of sensory coupling (sum of the mutual biases) depends on the experience of kinematic correlations between hand movements and cursor motions, whereas the asymmetry of sensory coupling (difference between the biases) depends on the relative reliabilities (inverse of variability) of hand-position and cursor-position estimates (reliability rule). Furthermore, the precision of movement control and perception of hand position are known to differ between hands (left, right) and workspaces (ipsilateral, contralateral), and so does the experience of kinematic correlations from daily life activities. Thus, in the present study, we tested whether strength and asymmetry of sensory coupling for the endpoints of reaches in a cursor control task differ between the right and left hand and between ipsilateral and contralateral hemispace. No differences were found in the strength of sensory coupling between hands or between hemispaces. However, asymmetry of sensory coupling was less in ipsilateral than in contralateral hemispace: in ipsilateral hemispace, the bias of the perceived hand position was reduced, which was accompanied by a smaller variability of the estimates. The variability of position estimates of the dominant right hand was also less than for the non-dominant left hand, but this difference was not accompanied by a difference in the asymmetry of sensory coupling – a violation of the reliability rule, probably due a stronger influence of visual information on right-hand movements. According to these results, the long-term effects of the experienced kinematic correlation between hand movements and cursor motions on the strength of sensory coupling are generic and not specific for hemispaces or hands, whereas the effects of relative reliabilities on the asymmetry of sensory coupling are specific for hemispaces but not for hands.

## Introduction

We perceive the world and our own body via different sensory modalities. The brain is thus faced with the challenge to form a unified percept from multiple signals. In particular, when the information provided by different modalities differs, it has to decide whether the discrepant information originates from a common source or from different sources. This process is known as causal inference (Körding et al., 2007; Wozny and Shams, 2011; Kayser and Shams, 2015; Rohe and Noppeney, 2015; Chen and Spence, 2017). Only with information that relates to a common source, such as proprioceptive and visual information on the position of the hand (Rossetti et al., 1995; Van Beers et al., 1999; Mikula et al., 2018), multisensory integration serves to enhance the precision of perception. However, partial multisensory integration, to which we refer as sensory coupling (e.g., Bresciani et al., 2006; Ernst, 2006), has also been observed for proprioceptive and visual information that relates to the positions of different and spatially separated objects and thus has different sources. This is the case in cursor-control tasks (controlling a cursor on a monitor by moving a hand held device) where proprioception refers to the position of the hand in the horizontal plane and vision to the position of the cursor on a roughly vertical monitor screen. In this type of task, estimates of discrepant positions of cursor and hand at the end of a movement, which result, for example, from a rotation of the cursor motion relative to the hand movement, are biased toward each other (Ladwig et al., 2012, 2013; Rand and Heuer, 2013, 2016, 2017, 2018; Kirsch et al., 2016, 2017; Debats et al., 2017a,b; Debats and Heuer, 2018a,b,c). According to Debats et al. (2017b), sensory coupling in a cursor-control task can be modeled as weighted average of the unisensory estimates and obeys the “reliability rule” (Colonius and Diederich, 2017), as is typical for multisensory integration where the weights in averaging the unisensory estimates are proportional to their relative reliabilities (e.g., Ernst and Bülthoff, 2004; Cheng et al., 2007). Here, we test whether the mutual biases of perceived positions of cursor and hand for a cursor-control reaching task depend on the specific movements when these differ both in their frequency in every-day life and in the reliability of perception and control. In particular, we test whether the respective differences between reaching movements in the ipsilateral and contralateral hemispace performed by the right and left hand are associated with different patterns of biases.

In the cursor-control task of the present study, the endpoints of reaching movements from a central start position into various directions are judged. Small and variable visual-feedback rotations serve to produce spatial discrepancies between the endpoints of cursor and hand. Sensory coupling then results in mutual biases of the respective judged positions toward each other. We express the biases as proportions of that spatial discrepancy, namely, of the visual-feedback rotation. Using these proportional biases, sensory coupling can be characterized in terms of its strength and asymmetry. Strength is measured as the sum of the proportional biases of hand-position and cursor-position judgments (Debats et al., 2017b). Coupling is perfect when the sum of the proportional biases is 1. In this case, the judgments of cursor position do not differ from the judgments of hand position. Asymmetry of sensory coupling is measured by the ratio of the proportional biases (cf. Debats et al., 2017b). However, this ratio becomes quite noisy when biases are only small. Therefore in the present study, we use the difference of the proportional biases of the judged hand and cursor positions as a measure of asymmetry. Typically, the bias of judged hand position toward the position of the cursor is stronger than the bias of the judged cursor position toward the position of the hand, so that the measure of asymmetry (hand-position bias minus cursor-position bias) is positive.

Regarding the strength of sensory coupling (i.e., the sum of biases of judged hand and cursor positions), we expect stronger coupling for the endpoints of movements in ipsilateral hemispace than for movements in contralateral hemispace, and for movements of the right hand than for movements of the left hand. These expectations are based on the following considerations: First, the strength of sensory coupling in the cursor-control task depends on the experience of kinematic correlations, that is, the systematic relations between hand movements and cursor motions with respect to kinematic variables such as velocity and direction (Debats et al., 2017a). These correlations, which determine the outcome of causal inference, can be varied experimentally, but they are also experienced in every-day life whenever a computer is used (Debats and Heuer, 2018b). More generally, causal inference and thus coupling strength is shaped at least to some degree by the long-term, pre-experimental cumulative experience of systematic relations between different sensory signals (cf. Ernst, 2007). The dependence of coupling strength on prior experience is consistent with the broad evidence on how perception in general is shaped by prior expectations (e.g., Körding and Wolpert, 2004; Knill, 2007; Waszak et al., 2012; Kumar and Mutha, 2016). Second, right-handers experience kinematic correlations of cursor motions and movements of the right hand, but only rarely, if at all, of the left hand. Similarly, but less obviously, movements in ipsilateral hemispace are more frequent than movements in contralateral hemispace (Howard et al., 2009). These considerations together suggest the hypothesis of a stronger sensory coupling for movements in ipsilateral than in contralateral hemispace, and for movements of the right than of the left hand because of differences in habitual use (cf. Dempsey-Jones and Kritikos, 2017) and thus different levels of experience of the relevant kinematic correlations.

Regarding the asymmetry of sensory coupling (i.e., the difference between biases of judged hand and cursor positions), we expect weaker asymmetry for movements in ipsilateral hemispace than for movements in contralateral hemispace, and for movements of the left hand than for movements of the right hand. According to the reliability rule, the hand-position bias should decline whenever the relative reliability of hand-position estimates is increased, thereby resulting in a decrease of the asymmetry. Thus, our expectation is based on the evidence of, first, more precise estimates of hand positions in ipsilateral than in contralateral hemispace and, second, more precise estimates of positions of the left hand than of positions of the right hand. With respect to hemispaces, the precision of proprioceptively sensed positions of the hand tends to be higher in ipsilateral than in contralateral hemispace (Bradshaw et al., 1983, 1989; Carson et al., 1990b; Imanaka et al., 1995; no differences have been reported by e.g., by Wilson et al., 2010). Ipsilateral reaches are also characterized by smaller errors, shorter movement times, and shorter deceleration times (e.g., Fisk and Goodale, 1985; Carson et al., 1990a; Mieschke et al., 2001; Kim et al., 2011; Carey and Liddle, 2013; Rand and Rentsch, 2017). With respect to hands, the position of the non-dominant hand tends to be judged more precisely than the position of the dominant hand, both in right-handers and in left-handers (Goble et al., 2006, 2009; Goble and Brown, 2007, 2008a; using the thumb: Roy and MacKenzie, 1978; Nishizawa and Saslow, 1987; Riolo-Quinn, 1991; no differences have been reported by e.g., Roy and MacKenzie, 1978; Carson et al., 1990b; Imanaka et al., 1995; Adamo and Martin, 2009; Wilson et al., 2010). This difference between the two hands could be related to different functions (cf. Goble and Brown, 2008b) and different control strategies (Annett et al., 1979; Haaland and Harrington, 1989; Bagesteiro and Sainburg, 2002; Wang and Sainburg, 2003, 2006; Sainburg and Schaefer, 2004; Sainburg, 2005).

Our expectation of different strengths of sensory coupling is based on the presupposition that causal inference in the cursor-control task is specific for each hand and each hemispace. Similarly, our expectation of different asymmetries of sensory coupling is based on the presupposition that estimates of relative sensory reliability are available and used specifically for each hand and hemispace. Alternatively, it is possible that these presuppositions may not hold. Instead, the characteristics of sensory coupling could be generalized across the specific movements performed in the service of a particular task, so that the strength and the relative weights are independent across those movements. In this case, we should find no variations of strength and asymmetry of sensory coupling across the different movements. Such possibility is suggested by recent findings of Mikula et al. (2018): when the visual information on the position of the hand was shifted by 10° by means of a prism, the bias of sensed hand position was not different between the left and right hand.

Biases of sensed hand and cursor positions can be assessed in different ways. In the present study, we ask our participants at the end of each trial to report the perceived position of the hand or the cursor at the end of the outward movement. They do so by matching the position of a visual marker to the remembered cursor position or by matching the position of the hand to the remembered hand position. We designate the resulting measures of the biases as *explicit* because participants are aware of reporting perceived positions. As in previous studies (Rand and Heuer, 2013, 2016, 2017, 2018; see also Mikula et al., 2018), we also use an additional implicit measure for the assessment of the bias of the sensed hand position. We designate this measure as *implicit* because the bias is inferred from the movements performed by the participants without them being aware of judging a position. The rationale of the measure is based on the finding that deviations of the sensed position of the hand from its actual position at the start of a movement result in systematic movement errors (cf. Bock and Eckmiller, 1986; Heuer and Sangals, 1998). In particular, such deviations can be induced by a discrepancy between proprioceptive and visual information on hand position, e.g., when the hand is seen through a prism (Rossetti et al., 1995; Mikula et al., 2018), when the other hand is seen in a mirror that is spatially displaced from the invisible hand used for the movement (Holmes et al., 2004; Holmes and Spence, 2005), or when a rubber hand or even a wooden block is seen in the mirror for about a dozen seconds (Holmes et al., 2006). In the present experiment, it is the final position of the outward movement of the hand and thus the start position of the return movement, for which perception should be biased by the deviating position of the cursor.

The reason for including the implicit measure of the bias of the sensed hand position is that in the past, we have identified some variables that affect the explicit and the implicit measure differently, e.g., aging (Rand and Heuer, 2013), a longer duration of the hand at the endpoint of the outward movement, which improves proprioceptive information (Rand and Heuer, 2016), a preceding adaptation to a visuomotor rotation (Rand and Heuer, 2017), and the proportion of trials in which the position of the hand or the cursor is judged (Rand and Heuer, 2018). These differences suggest that there might be two distinct representations of hand positions (e.g., Dijkerman and de Haan, 2007; De Vignemont, 2010), one serving perception (explicit) and the other one serving action or motor control (implicit). This would be similar to two distinct neural representations posited for vision (e.g., Milner and Goodale, 1995, 2008; Bridgeman et al., 1997). Here, we explored whether the explicit and implicit measures of the bias of sensed hand position might also be differently affected by the hand or the hemispace.

## Materials and Methods

### Participants

Twenty-eight right-handed healthy young adults [mean (SD) age: 23.9 (2.5) years, 14 males and 14 females] signed informed consent prior to participating in the study. Right-handedness was assessed by the Edinburgh Inventory (Oldfield, 1971). The mean (SD) laterality quotient was +84.9 (14.8). The study was conducted in accordance with the Declaration of Helsinki and with the approval by the ethics committee of the Leibniz Research Centre for Working Environment and Human Factors.

### Apparatus

**Figure 1:**
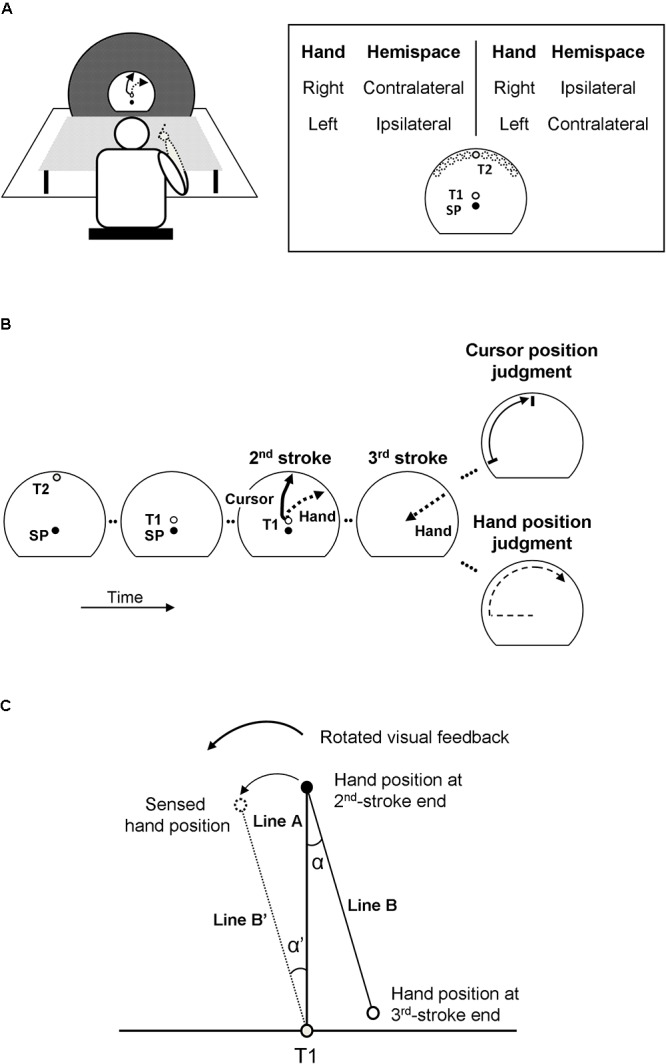
Behavioral task of a 3-stroke movement and analysis. **(A)** The experimental set up and target arrangements. SP, T1, and T2 refer to a starting position, a first target, and a second target, respectively. The locations of T2 presentation range between ±60° from the 12 o’clock position. The task was performed both with the dominant right hand and non-dominant left hand. Hemispaces (ipsilateral, contralateral) are defined relative to the hand used. **(B)** The judgment task of hand and cursor positions. The visual feedback of the 2nd-stroke is rotated and displayed simultaneously with hand movements. After the 2nd-stroke, the participants make a return movement without the visual feedback as the 3rd-stroke, and subsequently make an explicit judgment regarding the hand or cursor position. Arrows with solid line refer to motions of visual feedback (3rd panel) and a direction marker (5th panel, top). Arrows with dashed line refer to hand movements (not the visual feedback). **(C)** Implicit measure of hand position. The directional deviation α’ of the sensed hand position (dotted outline circle) from the physical one (black circle) at the end of the 2nd-stroke is estimated from the directional deviation α of the hand position at the end of the 3rd-stroke (solid outline circle) from its remembered target (T1, gray circle).

Participants sat at a table (Figure 1A), held a stylus with their right or left hand, and made three-stroke movements on a digitizer (Wacom Intuos 4 XL, 133 Hz sampling rate) in the horizontal plane while looking at a vertical monitor (Samsung SyncMaster 2233RZ, 22-inch, refresh rate 100 Hz). The center of the monitor and that of the digitizer were aligned with the midline of the participants. To reduce directional cues inherent to the normal rectangular shape of a monitor, it was covered by a large black circular screen with a semi-circular window of 32 cm diameter in its center. A first target (T1, 1.4 cm in diameter) was located in that center, and the start position (SP, 1.2 cm in diameter) was located 3 cm below T1. A second target (T2, 1 cm in diameter) was presented at pseudo random locations between -60° and +60° relative to the central location on an invisible circle with a radius of 15 cm around T1. When projected on the horizontal plane in which the movements were made, these targets were located in the ipsilateral or contralateral half of the workspace relative to the midsagittal plane of the participant that was aligned with T1. Corresponding to the invisible circle on the monitor, the workspace on the digitizer was bordered by a semi-circular plastic ring, the “stopper ring.” An opaque board placed above the participants’ arm blocked their direct view of the hand movements.

### Procedure

In two experimental conditions, participants used different hands to perform the movements (Figure 1A). The right-hand condition and the left-hand condition were tested in different sessions on different days, with their order being counterbalanced across participants. In each session, there were 144 experimental trials. In approximately half of the 144 trials (mean (SD): 72.09 (2.56) trials) movements were performed to targets in ipsilateral hemispace, and in the other half (71.91 (2.56) trials) to targets in contralateral hemispace (Figure 1A). During each session, experimental trials were organized in 4 blocks of 36 trials each (plus an initial warm-up trial). A short break was inserted after each block. In total, 288 trials were recorded for each participant. On the 1st day, the 144 experimental trials were preceded by 8 familiarization trials without and with the visual-feedback rotation and without a judgment; on both days, sessions started with 4 warm-up trials with visual-feedback rotation and judgment, two trials each for hand and cursor judgments.

At the beginning of each trial, participants were guided to the start position (SP) by arrows shown on the monitor. One second after the stylus was in the SP, the second target (T2) was presented for 1 s (Figure 1B, 1st panel). Subsequently, the first target (T1) appeared. After a delay of 0.5 s, an auditory go-signal was presented. The participants then made three-stroke movements at a comfortable speed. When T1 was reached, this target disappeared. Then, the participants made the 2nd stroke to the remembered T2 (Figure 1B, 3rd panel) until the movement hit the stopper ring. This movement we designate as the outward movement. Afterward, they made a return movement (3rd stroke) back to the remembered T1 location (Figure 1B, 4th panel). Note that the first stroke to T1 was introduced because the participants would naturally look at this target (Neggers and Bekkering, 2000; Rand, 2014), which prevented them from keeping their gaze on the position at which T2 had been presented as a strategy to remember that location.

The participants made the 1st and 2nd strokes with concurrent visual feedback provided by a cursor on the monitor, but the 3rd strokes (i.e., the return movements) were made without visual feedback. Only during the 2nd strokes (i.e., the outward movements from position T1 to the remembered position T2, Figure 1B, 3rd panel), the direction of cursor motion was rotated relative to the direction of hand movement by a randomly chosen angle out of 6 (clockwise rotation: -25°, -15°, -5°; counter-clockwise rotation: 5°, 15°, 25°). Among the 144 experimental trials of each session, each of the 6 visual-feedback rotations was presented in 24 trials.

One second after completion of the return movement (3rd stroke), participants were asked to judge either the hand or cursor position at the end of the outward movement. The judgment procedures were the same as those in our previous studies (Rand and Heuer, 2013, 2016, 2017, 2018). To indicate the type of judgment required, either the word “Hand” (for a judgment of hand position) or “Cursor” (for a judgment of cursor position) appeared briefly in the center of the monitor together with an arrow pointing to either the right (for a judgment using a counter-clockwise motion) or the left (for a judgment using a clockwise motion). For the judgment of cursor position (Figure 1B, 5th panel, top), a short line moved counter-clockwise or clockwise along the circular edge of the display window at a constant speed. This line passed all possible positions of the cursor at the end of the outward movement. The participant instructed the examiner to stop and finely adjust (back and/or forth) the line to the position that matched the remembered position. For the judgment of hand position (Figure 1B, 5th panel, bottom), the participant moved the pen from the right (or left) lower corner of the stopper ring counter-clockwise or clockwise along the ring and stopped where he/she judged the hand position to match the remembered position of the hand at the end of the outward movement. Note that motion of the visual marker and movement of the hand during the judgments differed from cursor motion and hand movement, respectively, during the outward movement (2nd stroke). Therefore, only positions on the circular path could be matched to remembered final positions of the outward movements, but not movement directions. The type of explicit judgment (hand or cursor position) was randomized across trials with the constraint of equal frequencies, and so was the direction of line or hand movement during the judgment (counter-clockwise or clockwise).

### Data Analysis

Judged and physical positions of cursor and hand at the end of the outward movements (2nd strokes) were measured in a polar coordinate system with the origin in position T1, the start position of the outward movements. Only the angles were analyzed because the distance from the origin was constant across all trials. For each trial, the angular deviation of the judged hand or cursor position from the respective physical position was determined (the counter-clockwise direction had a positive sign) and served as the explicit measure of the bias in that trial. Trials were sorted according to the four conditions of the experiment (Figure 1A), right-hand and left-hand trials (trials from separate sessions) as well as ipsilateral and contralateral trials (trials with movements in different directions). Ipsilateral trials were right-hand trials with target T2 to the right of the central location, and left-hand trials with target T2 to the left of the central location; contralateral trials were right-hand trials with target T2 to the left of the central location, and left-hand trials with target T2 to the right of the central location (Figure 1A).

Means and standard deviations of the angular deviations of the judged positions of cursor and hand from the respective physical positions were computed for each of the four experimental conditions, each of the six visual-feedback rotations, and each participant. The individual overall angular deviations, that is, the biases of the judged positions across all visual-feedback rotations, were computed for each experimental condition and judged position (cursor or hand) as the slopes of linear regressions of the angular deviations (dependent variable) on the visual-feedback rotations (independent variable) observed in the various trials. These slope parameters are estimates of the proportional biases. The intercept parameters of the linear regressions reflect the overall offsets of judgments that are unrelated to the visual-feedback rotations; they are reported as Supplementary Material. The individual overall variabilities of the angular deviations were computed as the mean standard deviations across all visual-feedback rotations for each experimental condition and judged position (cursor or hand).

The implicit measure of the bias of the sensed hand position toward the position of the cursor in each trial was the angular deviation of the direction of the return movement (3rd stroke) from the direction of the outward movement (2nd stroke). This deviation is illustrated as α’ = α in Figure 1C. The counter-clockwise direction of the angular deviation α’ had a positive sign. Individual means and standard deviations of the implicit measure were computed for each experimental condition and each visual-feedback rotation across all trials with cursor-position and hand-position judgments because the return movements were made before the type of judgment was instructed. The angular deviations α’ of all trials of each experimental condition were subjected to the same linear regressions as the angular deviations of the explicit judgments of hand positions from the physical positions. The slope parameters of the regressions served as overall measures of the implicitly assessed proportional biases of sensed hand position toward the position of the cursor. The intercept parameters measured the overall offsets of implicitly assessed hand positions unrelated to the rotation (or the constant errors of the return movements); they are reported as Supplementary Material. The individual overall variabilities of the implicitly assessed biases were computed for each experimental condition as the mean standard deviation across all visual-feedback rotations.

The data were screened for outliers both among trials and among participants. Based on the linear regressions computed separately for each type of bias assessment (cursor-explicit, hand-explicit, hand-implicit), each experimental condition, and each participant, trials with angular deviations outside the range of predicted deviations ± 3 standard deviations of the residuals were eliminated as outliers among trials. In total, 58 out of 8064 experimental trials (0.72%) were removed from all analyses. Subsequently, the slope parameters (proportional biases) for each type of assessment and each condition were screened for outliers among participants. Means and standard deviations across all participants were calculated for the three types of measurement and the four experimental conditions, and slope parameters outside the range of mean ± 3 standard deviations were defined as outliers. These computations were repeated until no further outliers were found. As the result, one participant was identified as having outliers for the explicitly assessed bias of cursor position and was excluded from all analyses.

The individual explicitly assessed proportional biases of hand and cursor judgments were subjected to a 2 (hand: right, left) × 2 (hemispace: ipsilateral, contralateral) × 2 (type of measure: hand-explicit, cursor-explicit) repeated-measures ANOVA. In this ANOVA, main effects of hand and hemispace as well as the interaction of these two factors indicate differences between conditions in coupling strength. More precisely, each of these main effects indicates a difference between the means of the biases of cursor-position and hand-position judgments. Since coupling strength is defined as the sum of the biases of the two types of judgments, and since these means are half the coupling strengths in the four conditions (2 hands × 2 hemispaces), they suffice to represent coupling strength in these statistical analyses. In contrast, the main effect of type of measure reflects the asymmetry of sensory coupling (i.e., the difference between the biases of hand-position and cursor-position judgments). Any interaction of this factor with hand or hemispace indicates a modulation of the asymmetry across hands and/or hemispaces.

We ran the same type of 2 (hand) × 2 (hemispace) × 2 (type of measure: hand-explicit, hand-implicit) ANOVA for the explicitly and implicitly assessed biases of sensed hand position. Here the main effects of hand and hemispace as well as the interaction of these two factors indicate common variations of explicitly and implicitly assessed biases of sensed hand position. The main effect of type of measure captures the difference between them, and any interaction of this factor with hand or hemispace indicates a modulation of the difference, that is, contrasting effects of hand or hemispace on the explicitly and implicitly assessed biases of sensed hand position. The individual standard deviations (i.e., overall intra-individual variabilities) were subjected to corresponding ANOVAs.

## Results

We first report the findings on the explicit measures of the biases of sensed hand and cursor positions, followed by the analysis of the intra-individual variability of these measures. Subsequently, we turn to the comparison of the explicit and implicit measures of the bias of sensed hand position and their variability.

### Explicit Measures of the Biases of Sensed Cursor and Hand Positions

**Figure 2:**
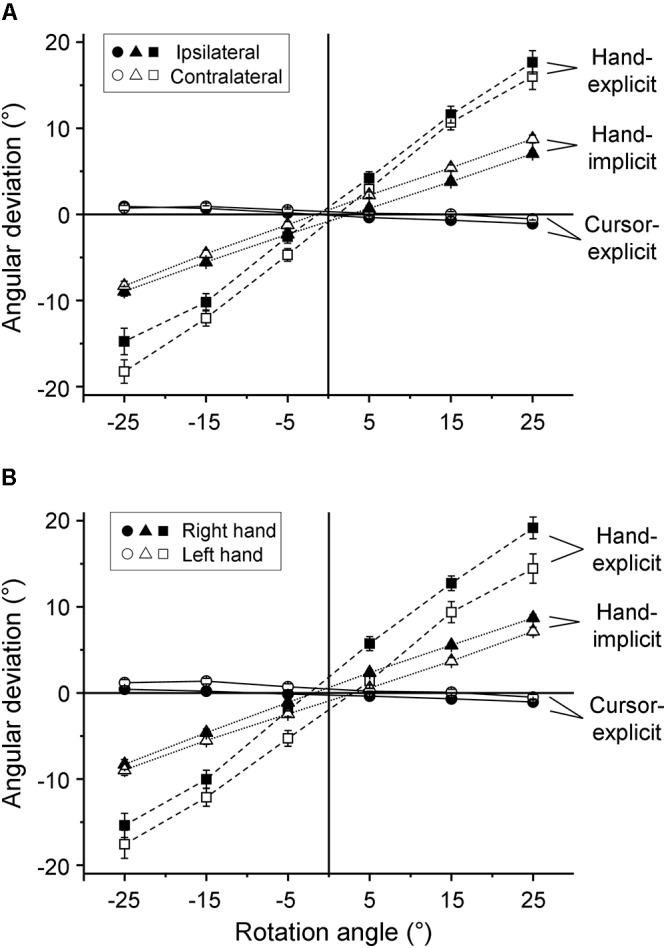
Explicit and implicit judgments. Mean angular deviations of the judged directions from the corresponding physical directions as a function of the rotation of visual feedback. **(A)** Differences between the ipsilateral (filled symbols) and contralateral (open symbols) hemispaces are shown while values of the right and left hands are pooled. **(B)** Differences between the right (filled symbols) and left (open symbols) hands are shown, while values of ipsilateral and contralateral hemispaces are pooled. The mean values across all participants are plotted for explicit measure of hand position (squares), explicit measure of cursor position (circles), and implicit measure of hand position (triangles). The error bars represent the SE.

The mean explicitly assessed angular deviations of the sensed hand and cursor positions from the respective physical positions of hand and cursor are depicted in Figure 2 as a function of the visual-feedback rotation. Figure 2A is for comparisons between the ipsilateral and contralateral hemispaces, and Figure 2B between the right and left hands. The mean biases of the judged hand position showed steep positive slopes (Figure 2A,B, hand-explicit), indicating a strong proportional bias toward the position of the cursor. In contrast, the mean biases of the judged cursor position showed slightly negative slopes (Figure 2A,B, cursor-explicit), indicating a weak proportional bias of the judgments of cursor position toward the position of the hand. The slopes for hand-position judgments were obviously different from zero. Therefore, we tested the individual slopes against zero only for the cursor-position judgments. One-sample *t*-tests turned out to be significant for each of the four experimental conditions (right hand-ipsilateral: *t*(26) = 2.32, *p* = 0.028; right hand-contralateral: *t*(26) = 2.08, *p* = 0.048; left hand-ipsilateral: *t*(26) = 3.94, *p* = 0.001; left hand-contralateral: *t*(26) = 2.35, *p* = 0.027).

**Figure 3:**
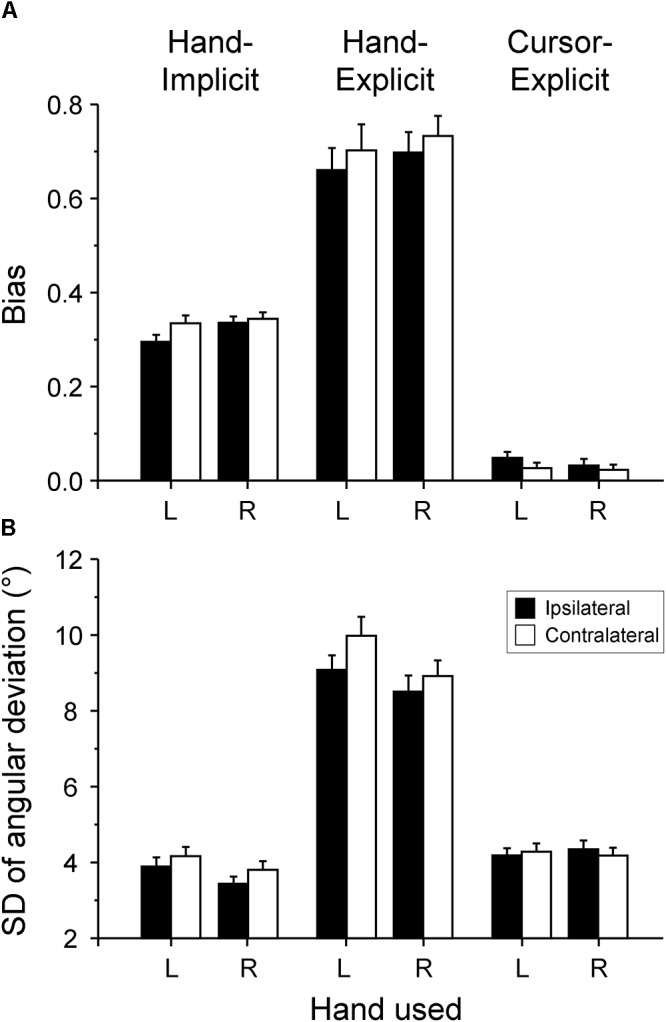
Proportional biases and intra-individual variability of sensed hand and cursor positions. **(A)** Mean proportional biases of explicitly measured hand position (hand-explicit) and cursor position (cursor-explicit), and implicitly measured hand position (hand-implicit). **(B)** Mean overall standard deviations (SD) of the angular deviations of the judged directions from the corresponding physical directions. Mean values across participants are plotted for the ipsilateral (filled columns) and contralateral (open columns) hemispace and for the right (R) and left (L) hand. The error bars represent the SE.

For both types of explicit measure (hand-explicit and cursor-explicit), the means of the individual proportional biases are plotted in Figure 3A. As the biases of judged hand and cursor positions were in opposite directions relative to the visual-feedback rotation, the biases of the judged cursor position were multiplied by -1. Thus, in the following, positive biases of judged cursor positions are biases toward the position of the hand, and positive biases of judged hand position are biases toward the position of the cursor.

The first focus of this study is on the effects of hand and hemispace on coupling strength, which is the sum of the explicitly assessed proportional biases of the hand-position and cursor-position judgments. The mean (SE) coupling strengths were 0.730 (0.041) and 0.756 (0.043) in ipsilateral and contralateral hemispace, respectively, for the right hand. The corresponding coupling strengths for the left hand were 0.709 (0.049) and 0.729 (0.058). According to the 2 (hand: right, left) × 2 (hemispace: ipsilateral, contralateral) × 2 (type of measure: hand-explicit, cursor-explicit) ANOVA, the main effects of hand [*F*(1,26) = 0.48, *p* = 0.496, $\eta_{p}^{2}$ = 0.018] and hemispace [*F*(1,26) = 0.86, *p* = 0.363, $\eta_{p}^{2}$ = 0.032] were not significant. The above ANOVA also revealed no significant interaction of hand and hemispace [*F*(1,26) = 0.02, *p* = 0.905, $\eta_{p}^{2}$ = 0.001]. Thus, there was no reliable variation of the strength of sensory coupling across the four experimental conditions.

The second focus of this study is on the effects of hand and hemispace on the asymmetry of sensory coupling. The mean bias of judged hand position toward the cursor position was weaker in ipsilateral hemispace than in contralateral hemispace (Figure 3A, hand-explicit), whereas the mean bias of judged cursor position toward the hand position was stronger in ipsilateral than in contralateral hemispace (Figure 3A, cursor-explicit). The mean (SE) differences between the biases of judged hand and cursor positions, that is, the measures of the asymmetry of sensory coupling, were 0.665 (0.051) and 0.710 (0.045) in ipsilateral and contralateral hemispace, respectively, for the right hand. The corresponding coupling asymmetry for the left hand were 0.611 (0.049) and 0.675 (0.055). The ANOVA first revealed a reliable main effect of type of measure, that is, a reliable asymmetry of sensory coupling overall: the bias of judged hand position toward the cursor position was significantly stronger than the bias of judged cursor position toward the hand position [*F*(1,26) = 223.56, *p* < 0.001, $\eta_{p}^{2}$ = 0.896]. More importantly, the interaction of type of measure with hemispace was significant, indicating a reliable difference of the asymmetry between hemispaces [*F*(1,26) = 5.62, *p* = 0.025, $\eta_{p}^{2}$ = *0.178*]. In contrast, the interaction of type of measure and hand was not significant [*F*(1,26) = 1.87, *p* = 0.183, $\eta_{p}^{2}$ = 0.067], indicating no reliable effect of the hand on the asymmetry of sensory coupling.

### Intra-Individual Variability of Explicitly Assessed Biases

The mean intra-individual standard deviations of the explicitly assessed biases of hand and cursor positions are shown in Figure 3B. The mean variability of the biases of the judged cursor position (Figure 3B, cursor-explicit) was substantially smaller than that of the judged hand position (Figure 3B, hand-explicit). According to the 2 × 2 × 2 ANOVA, the main effect of type of measure was significant [*F*(1,26) = 181.83, *p* < 0.001, $\eta_{p}^{2}$ = 0.875]. Whereas the variability of the bias of judged cursor position was essentially the same across the four experimental conditions (Figure 3B, cursor-explicit), the variability of the bias of judged hand position varied (Figure 3B, hand-explicit). First, the mean standard deviation of the bias of judged hand position was smaller in ipsilateral than in contralateral hemispace, giving rise to a significant interaction of hemispace and type of measure [*F*(1,26) = 5.40, *p* = 0.028, $\eta_{p}^{2}$ = 0.172] and an almost significant main effect of hemispace [*F*(1,26) = 3.50, *p* = 0.073, $\eta_{p}^{2}$ = 0.119]. Second, the mean standard deviation was smaller for the right hand than for the left hand, giving rise to a significant interaction of hand and type of measure [*F*(1,26) = 8.85, *p* = 0.006, $\eta_{p}^{2}$ = 0.254] and a significant main effect of hand [*F*(1,26) = 5.43, *p* = 0.028, $\eta_{p}^{2}$ = 0.173].

### Explicit and Implicit Measures of the Bias of Sensed Hand Position and Their Variability

In a second series of analyses, we compared the explicit measure of the bias of sensed hand position toward the position of the cursor with the implicit measure. The mean angular deviations of the directions of the return movements from the directions of the outward movements had positive slopes as a function of the visual-feedback rotation (Figure 2A,B, hand-implicit), but the slopes were less steep than those of the explicit measure (Figure 2A,B, hand-explicit). Thus, there was a modest implicitly assessed proportional bias of the sensed hand position toward the position of the cursor that was weaker than the explicitly assessed bias.

Figure 3A shows the means of the individual proportional biases (slopes of the linear regressions) of both explicit and implicit measures for each of the four experimental conditions. According to the 2 (hand: right, left) × 2 (hemispace: ipsilateral, contralateral) × 2 (type of measure: hand-implicit, hand-explicit) ANOVA, the implicitly assessed biases were significantly smaller than the explicitly assessed biases [*F*(1,26) = 69.43, *p* < 0.001, $\eta_{p}^{2}$ = 0.728]. Regarding the four experimental conditions, the biases in ipsilateral hemispace were significantly smaller than the biases in contralateral hemispace [*F*(1,26) = 7.87, *p* = 0.009, $\eta_{p}^{2}$ = 0.232]. In addition, the dominant right hand tended to have stronger biases than the non-dominant left hand; however, this difference was not significant [*F*(1,26) = 3.19, *p* = 0.086, $\eta_{p}^{2}$ = 0.109]. There was no statistically significant interaction. Thus, there was no indication of a modulation of the difference between the explicitly and the implicitly assessed biases by hand or hemispace.

As shown in Figure 3B, the mean intra-individual standard deviations of the implicit measure of the bias of sensed hand position toward the position of the cursor were substantially smaller than those of the explicit measure. According to the 2 × 2 × 2 ANOVA, the main effect of type of measure was significant [*F*(1,26) = 201.82, *p* < 0.001, $\eta_{p}^{2}$ = 0.886]. Intra-individual variability was significantly smaller for the ipsilateral hemispace than for the contralateral hemispace [*F*(1,26) = 9.77, *p* = 0.004, $\eta_{p}^{2}$ = 0.273], and it was significantly smaller for the dominant right hand than for the non-dominant left hand [*F*(1,26) = 14.78, *p* = 0.001, $\eta_{p}^{2}$ = 0.363]. However, interactions, in particular interactions with the type of measure, were not significant. Thus, the difference between the mean standard deviations of the explicitly and implicitly assessed biases was not modulated by the experimental conditions.

## Discussion

The main results of the present study are: (1) the strength of sensory coupling does not differ between ipsilateral and contralateral hemispace, but the asymmetry of sensory coupling is weaker in ipsilateral hemispace; (2) the weaker asymmetry of sensory coupling in ipsilateral hemispace is accompanied by smaller variability of the bias of sensed hand position; (3) neither the strength nor the asymmetry of sensory coupling differ between the dominant and non-dominant hand; (4) although sensory coupling is not reliably different between the two hands, intra-individual variability of the bias of sensed hand position is smaller in the dominant than in the non-dominant hand; (5) implicit and explicit measures of the bias of sensed hand position toward the position of the cursor are not differently affected by hemispace and hand. We discuss the findings on hemispaces, hands, and implicit and explicit measures in turn.

### Ipsilateral and Contralateral Hemispaces

Sensory coupling is less asymmetric for the end positions of movements into ipsilateral hemispace than for those into contralateral hemispace. The weaker asymmetry reflects a relatively stronger weight of the sensed hand position in sensory coupling. It is accompanied by smaller intra-individual variability and thus higher reliability of the judgments of hand position in ipsilateral than in contralateral hemispace. Hence, the variation in sensory coupling across the two hemispaces is consistent with the reliability rule (Colonius and Diederich, 2017), according to which the sensory modality with higher reliability is weighted more heavily in integrating estimates based on different sensory modalities.

More precisely, the reliability rule holds for variabilities of unisensory estimates, which were not assessed in the present study. However, in other studies of sensory coupling in cursor-control tasks (Debats et al., 2017a,b; Debats and Heuer, 2018a,b,c), reliability was assessed in unimodal trials in which hand movements were made with no visual feedback and cursor motions were seen without performing hand movements. In these studies, the variations of reliability measured in unimodal trials were generally paralleled by (somewhat attenuated) variations of reliability measured in bimodal trials. Thus, the different variabilities observed in bimodal trials of the present experiment in the two hemispaces should indeed reflect variations of unisensory reliabilities, in particular since the strength of sensory coupling was the same for both hemispaces (cf. Debats et al., 2017b). The observed difference between hemispaces is also consistent with other studies showing higher precision of proprioception-based position judgments in ipsilateral hemispace (Bradshaw et al., 1983, 1989; Carson et al., 1990b; Imanaka et al., 1995) as well as observations of superior sensorimotor control of ipsilateral reaches compared with contralateral reaches (Fisk and Goodale, 1985; Carson et al., 1990a; Mieschke et al., 2001; Kim et al., 2011; Carey and Liddle, 2013; Rand and Rentsch, 2017).

We found no indication of a stronger sensory coupling in ipsilateral than in contralateral hemispace. Such difference would be expected from the hypothesis that multisensory integration is strongest in “habitual action space” (Dempsey-Jones and Kritikos, 2017), which is the ipsilateral hemispace rather than the contralateral one for each hand (Howard et al., 2009). The everyday experience of cursor control in using computers is obviously more frequent in ipsilateral hemispace (and with the dominant hand). The lack of a corresponding difference in coupling strength between hemispaces can be taken to suggest that the effects of cumulative everyday experience on the strength of sensory coupling generalize across hemispaces. In other words, the strength of sensory coupling is shaped by the everyday experience in a generalized way, probably for a particular type of task irrespective of the region of the workspace where it is performed (cf. Mikula et al., 2018). However, this conclusion is tentative at present as we cannot exclude that different effects of cumulative experience in the two hemispaces could have been masked by the effects of the immediate experience of the systematic relations between hand and cursor movements in each trial. In the present study, there was continuous visual feedback in each individual trial that enhances the strength of sensory coupling (cf. Debats and Heuer, 2018b) and could possibly compensate eventual differences due to different levels of preceding experience. If this assumption were correct, a difference in the strength of sensory coupling between hemispaces should appear with terminal (endpoint) visual feedback in each trial rather than continuous feedback as used in the present study.

### Dominant and Non-dominant Hands

The intra-individual variability of the bias of sensed hand position was found to be smaller in the dominant right hand than in the non-dominant left hand. Thus, the dominant hand had superior precision of hand-position judgments when bimodal (visual and proprioceptive) stimuli were presented. Unlike the difference between ipsilateral and contralateral hemispaces, the higher precision of judged hand positions for the dominant right hand was not accompanied by a reduced asymmetry of sensory coupling with a weaker bias of sensed hand position toward the position of the cursor. This would have been expected by the reliability rule (Colonius and Diederich, 2017). Instead, if anything, the bias for the right hand tended to be stronger than for the left hand, both for the explicit and the implicit measure. Thus, the reliability rule was clearly violated.

There are reasons to reckon a stronger influence of visual information for the dominant right hand than for the non-dominant left hand, which could result in a violation of the reliability rule which does not always strictly hold in the cursor-control task (cf. Debats et al., 2017b). For example, in a previous study (Rand and Heuer, 2013), we found differences between young and older adults similar to those between the left and right hand in the present study. In that study, a significantly smaller variability of the judged hand position in older adults was accompanied by a significantly stronger bias of the judged hand position. This is a clear violation of the reliability rule which would have predicted a stronger weight of proprioceptive information and thus a weaker bias. However, a stronger bias of the judged hand position and thus a relatively stronger weight of visual information in sensory coupling is in agreement with the observation that older adults are more dependent on visual feedback during goal-directed actions (Haaland et al., 1993; Seidler-Dobrin and Stelmach, 1998; Simoneau et al., 1999).

Regarding differences between hands, Goble and Brown (2008a) showed a higher precision of the dominant right hand compared to the non-dominant hand in matching forearm positions to visual targets, whereas the difference between hands becomes the opposite in the case of proprioceptive targets. This again suggests a particularly strong influence of visual information on the felt position of the dominant hand. Wilson et al. (2010) showed a right-hand superiority in proprioceptive judgments of hand positions relative to visually presented targets, but not to proprioceptive targets. This hand difference was observed only in the central region of the workspace where object manipulations occur frequently. Finally, the allocation of visual attention is known to be biased toward objects that appear in the space around the hand (Reed et al., 2006; Tseng et al., 2012), especially for the dominant hand for right-handers (Le Bigot and Grosjean, 2012, 2016). The strong influence of visual information on perception and action of the dominant right hand is probably related to functional differences between the hands in everyday activities. Namely, the dominant right hand is preferably used for object manipulations with visual feedback while the non-dominant left hand is used for object stabilization (Guiard, 1987; Goble and Brown, 2008a,b).

Regarding the strength of sensory coupling, according to the notion of habitual action space (Dempsey-Jones and Kritikos, 2017), one would expect that the cumulative experience of the systematic relation between hand movements and cursor motions in cursor-control tasks results in a stronger sensory coupling for the dominant right hand. From that perspective, the difference between hands should probably be even larger than the difference between hemispaces. However, there was at the most a tendency toward such a difference, but no reliable effect. Similar to the absence of such a difference between ipsilateral and contralateral hemispace, the effects of cumulative experience could have generalized across hands (Mikula et al., 2018). Alternatively, the difference between hands could have been masked by the immediate experience of the relation between hand movements and cursor motions in each individual trial.

### Explicit and Implicit Measures

Explicit and implicit measures of the bias of sensed hand position toward the position of the cursor were similarly influenced by hand and hemispace. These findings add to a number of shared and distinctive properties of the implicit and explicit measures that we have previously observed (Rand and Heuer, 2013, 2016, 2017, 2018; Rand and Shimansky, 2018). The differences in the magnitude of the bias and the intra-individual variability of explicit and implicit measures were consistently found in previous studies (Rand and Heuer, 2013, 2016, 2017, 2018) and the present one. More importantly, the two types of measure were differently affected by aging, visuo-motor adaptation, additional proprioceptive information at the end of the outward movement, and the relative frequency of trials with explicit judgments of cursor and hand positions (Rand and Heuer, 2013, 2016, 2017, 2018). On the other hand, similarities between the two types of measure were observed when the predictability of trials with hand-position and cursor-position judgments was varied or the homogeneity of these trial types within blocks of trials (Rand and Heuer, 2016). Also when the relative frequencies of both types of trials were quite different, namely 80 % and 20 %, both measures similarly reflected relatively stronger weight of the more frequently relevant modality between vision and proprioception (Rand and Heuer, 2018). The current study extends the list of shared properties by parallel effects of hand and hemispace.

## Conclusion

In the present study, we found no differences between hemispaces and hands with respect to the strength of sensory coupling in a cursor-control task in spite of different levels of everyday-experience of cursor-control. This suggests that the effects of everyday experience are generic, probably related to cursor-control in general, and not specific for the one or the other hand or different regions of the workspace (cf. Mikula et al., 2018). In contrast, we found a weaker asymmetry of sensory coupling in ipsilateral than in contralateral hemispace. This difference is consistent with reliability-based weighting in sensory integration: for ipsilateral reaches, there is a stronger weight of the comparatively more reliable proprioceptive hand-position information and a correspondingly weaker weight of visual cursor-position information. Differences in the asymmetry between the dominant and non-dominant hand were less clear-cut. Most likely there is a stronger influence of visual information for the dominant hand, which results in a stronger weight of vision in sensory integration than expected from reliability-based weighting.

## Author Contributions

MR and HH conceived and designed the experiments, analyzed the data, and wrote the paper. MR performed the experiments.

## Conflict of Interest Statement

The authors declare that the research was conducted in the absence of any commercial or financial relationships that could be construed as a potential conflict of interest.
