# Supplementary material for: Effects of Hand and Hemispace on Multisensory Integration of Hand Position and Visual Feedback
Source: Front Psychol. 2019 Feb 12;10:237. doi: 10.3389/fpsyg.2019.00237 (PMC6379332; doi:10.3389/fpsyg.2019.00237)
Supplement: Supplementary file 1 [file Data_Sheet_1.pdf]

Supplementary Material

Effects of hand and hemispace on multisensory integration of hand position and visual feedback

Miya K. Rand*, Herbert Heuer
*** Correspondence:**
Miya K. Rand:
rand@ifado.de

1 Intercepts of the linear regressions of angular deviations on visual-feedback rotations.

For each of the three bias measures (explicitly measured hand position and cursor position and implicitly measured hand position, Fig. 2a, b), the linear regressions of angular deviations on visual feedback rotations yielded not only slopes (reported in Fig. 3a), but also intercepts. These intercepts are unrelated to sensory coupling, but reflect overall offsets of the judgments of hand or cursor positions in the counter-clockwise (CCW) or clockwise (CW) direction independent of the visual-feedback rotation. Supplementary Figure 1 shows the mean intercepts for the three types of measure. They are plotted separately for the right and left hemispaces because they were more consistently different between the right and left than between the ipsilateral and contralateral hemispaces.

In terms of the explicitly measured sensed hand and cursor positions (Suppl. Fig. 1, hand-explicit, cursor-explicit), the intercepts for the left (or right) hemispace were consistently positive (or negative), indicating that the participants generally judged their hand and cursor positions to be slightly rotated in the CCW (or CW) direction compared to the actual position, regardless of the hand used. In other words, the error was systematic, specifically a bias toward the horizontal (on the monitor) or the frontoparallel plane (on the digitizer). In a 2 (type of measure: hand explicit, cursor explicit) x 2 (hand: right, left) x 2 (hemispace: right, left) ANOVA, these systematic errors gave rise to a significant main effect of hemispace ($F(1,26) = 67.14$, $p < 0.001$, $\eta_p^2 = 0.721$). The basic difference between the right and left hemispace was modulated in several ways. It was greater for the left hand compared to the right hand (hand x hemispace interaction: $F(1,26) = 5.10$, $p = 0.032$, $\eta_p^2 = 0.164$), it was greater for hand judgments than for cursor judgments (type of measure x hemispace interaction: $F(1,26) = 21.06$, $p < 0.001$, $\eta_p^2 = 0.447$), and the difference between the two hands was essentially restricted to hand judgments and absent or even reversed for cursor judgments (3-way interaction: $F(1,26) = 8.34$, $p = 0.008$, $\eta_p^2 = 0.243$). For hand judgments, but not for cursor judgments, the errors were systematically more positive (CCW) for the right than for the left hand, giving rise to a significant main effect of hand ($F(1,26) = 7.30$, $p = 0.012$, $\eta_p^2 = 0.219$) and a significant interaction of type of measure and hand ($F(1,26) = 20.25$, $p < 0.001$, $\eta_p^2 = 0.438$).

For the implicitly-measured sensed hand position (Suppl. Fig. 1, hand-implicit), the intercepts showed opposite systematic errors in the left and right hemispace as compared to the explicitly measured sensed hand position. Namely, for the left (or right) hemispace, errors of sensed hand positions were in the CW (or CCW) direction compared to the actual position. Thus, the errors were toward a sagittal plane rather than a frontoparallel plane. Consequently, a 2 (type of measure: hand implicit, hand explicit) x 2 (hand: right, left) x 2 (hemispace: right, left) ANOVA revealed a

significant hemispace effect (F(1,26) = 9.13, p = 0.006, η_p^2 = 0.260), which was greater for the explicit than for the implicit type of measure (type of measure x hemispace interaction: F(1,26) = 52.65, $p < 0.001$, η_p^2 = 0.669). In absolute terms, the difference between hemispaces was greater for the left than for the right hand (3-way interaction: F(1,26) = 14.93, p = 0.001, η_p^2 = 0.365; this modulation resulted in the 3-way interaction because the differences between hemispaces were negative and positive for the implicit and explicit measures, respectively). Finally, for the right hand, errors were systematically more positive (CCW) than for the left hand (F(1,26) = 29.46, $p < 0.001$, η_p^2 = 0.531).

2 Supplementary Figure.

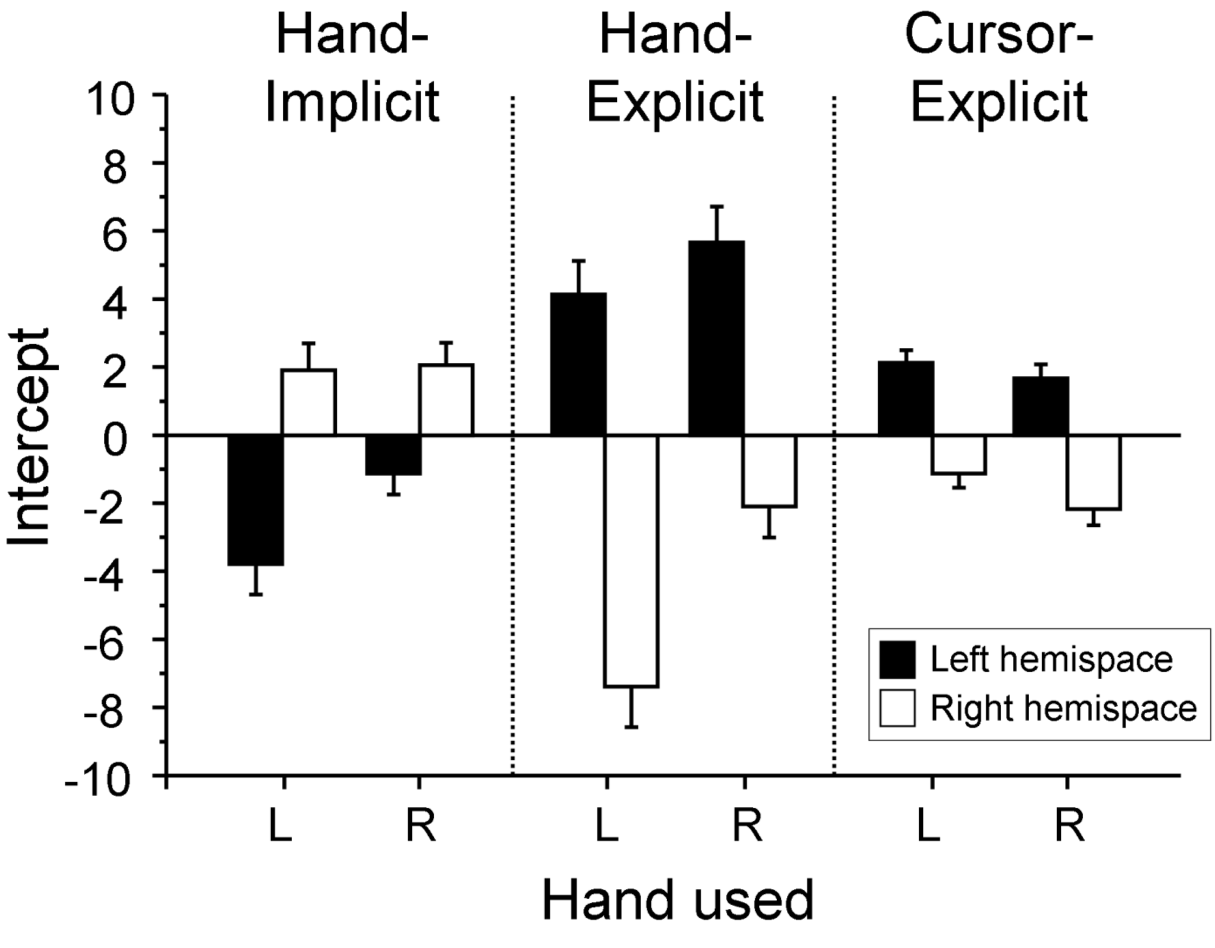

Supplementary Figure 1. Mean intercepts of explicitly measured hand position (hand-explicit) and cursor position (cursor-explicit), and implicitly measured hand position (hand-implicit). Mean values across participants are plotted for the right (open columns) and left (filled columns) hemispace and for the right (R) and left (L) hand. The error bars represent the SE.
